# ST-segment elevation predicts the occurrence of malignant ventricular arrhythmia events in patients with acute ST-segment elevation myocardial infarction

**DOI:** 10.1186/s12872-023-03099-w

**Published:** 2023-02-02

**Authors:** Xianpei Wang, Lifang Wei, Ying Wu, Juanjuan Yan, Linwei Zhao, Xinjie Yue, Chuanyu Gao

**Affiliations:** 1 Department of Cardiology, People’s Hospital of Zhengzhou University, Henan Provincial People’s Hospital, Zhengzhou University Central China Fuwai Hospital, No. 1 Fuwai Avenue, Zhengdong New District, Zhengzhou, Henan Province China; 2 Henan Provincial Key Lab for Control of Coronary Heart Disease, Zhengzhou University Central China Fuwai Hospital, People’s Hospital of Zhengzhou University, Zhengzhou, Henan Province China

**Keywords:** ST-segment elevation, Malignant ventricular arrhythmia events, Electrocardiogram, Myocardial infarction, Percutaneous coronary intervention

## Abstract

**Background:**

ST-segment elevation (STE) represents a repolarization dispersion marker underlying arrhythmogenesis in ST-segment elevation myocardial infarction (STEMI); however, its value for predicting malignant ventricular arrhythmia events (MVAEs) remains uncertain.

**Methods:**

In total, 285 patients with STEMI and those with or without MVAEs who presented within 6 h of symptom onset were enrolled. The relationships between STE and clinical characteristics of MVAEs (defined as ventricular tachycardia or ventricular fibrillation) were analyzed using t-test, chi-square test, binary multivariate logistic regression, and receiver operating characteristic curve analysis.

**Results:**

Patients with STEMI and MVAEs had a shorter time from symptom onset to balloon time (*p* = 0.0285) and greater STE (*p* < 0.01) than those without MVAEs. The symptom-to-balloon time, age, and STE were associated with MVAEs after stepwise regression analysis in all cases. Only STE was significantly associated with the occurrence of MVAEs (all, *p* < 0.01). The area under the curve (AUC) of STE for predicting MVAEs was 0.905, and the cut-off value was 4.5 mV. When only infarct-related arteries were included in the analysis, the AUC of the left anterior descending artery was 0.925 with a cut-off value of 4.5 mV, that of the right coronary artery was 0.915 with a cut-off value of 4.5 mV, and that of the left circumflex artery was 0.929 with a cut-off value of 4.0 mV.

**Conclusions:**

In patients with STEMI presenting within 6 h of symptom onset, age, symptom-to-balloon time, and STE were the main predictors for MVAEs. However, among these, STE was the strongest predictor for MVAEs and was an index for repolarization dispersion of cardiomyocytes in infarcted and non-infarcted areas.

## Introduction

Coronary artery disease, especially myocardial infarction (MI), is believed to account for 75% of sudden cardiac deaths (SCDs). It is also estimated that SCD accounts for 30–50% of all coronary deaths [1, 2]. Up to 20% of out-of-hospital cardiac arrests due to malignant ventricular tachyarrhythmia, including ventricular tachycardia (VT) and ventricular fibrillation (VF), develop in the acute phase of MI [3]. In patients with ST-segment elevation myocardial infarction (STEMI), the incidence of early VT/VF was 3.0% [4]; however, STEMI has a significant impact on prognosis and can lead to death. Although many indexes are related to the occurrence of malignant arrhythmia, VT/VF or sudden death is still difficult to predict. Some studies have shown that in STEMI, the type and shape of ST-segment elevation (STE) can predict the occurrence of VT/VF although its incidence in electrocardiography (ECG) pattern is only 1.4% [5].

Our study involved patients with STE and variant angina pectoris and found that a more elevated ST segment and prolonged interval from the peak to the end of the T wave (Tp-e interval) can predict the occurrence of malignant arrhythmias [6, 7]. Because variant angina has the characteristic ischemia that can be completely reversed, it can be used as a model to study the relationship between STE and MVAEs. Moreover, our study showed that in patients with STEMI undergoing primary percutaneous coronary intervention (PCI), the duration of ST-segment resolution (STR) can predict malignant arrhythmia and long-term prognosis, and STE is related to the electrophysiological mechanism of the prolonged Tp-e interval [8]. Other studies have suggested that a prolonged Tp-e interval and elevated ST segment amplitude are independent risk factors for reperfusion VF in unselected patients with STEMI [9, 10].

However, in the out-of-hospital setting, emergency department, and catheter room, the immeasurability of the Tp-e interval and the variability of STE in the very early stage of STEMI necessitates reevaluation of simpler and more sensitive indicators to predict MVAEs. Therefore, the present study aimed to analyze the ECG characteristics associated with MVAEs during pre-reperfusion and periprocedural reperfusion in selected patients with early STEMI treated with PCI.

## Methods

Patients were recruited from the database of People's Hospital of Zhengzhou University and Fuwai Central China Cardiovascular Hospital (Zhengzhou, China) between January 2017 and June 2020. Overall, 301 patients with the first episode of STEMI seen < 6 h after the onset of symptoms were retrospectively assessed if they had the following: typical chest pain lasting > 30 min, ECG pattern showing ≥ 0.1 mV ST-segment elevation in ≥ 2 limb leads or ≥ 0.2 mV STE in two contiguous precordial leads, and elevated serum troponin I levels greater than the upper limit of normal. All patients underwent angiography and showed confirmatory angiographic evidence of total occlusion or recanalization with fresh thrombus, that is, thrombolysis in myocardial infarction (TIMI) grade 0–3 flow of the infarct-related coronary artery. All patients who underwent PCI and had restored TIMI grade 3 flow were included in the study. The exclusion criteria were as follows: patients who had a previous history of angina pectoris or MI, left or right bundle branch block on ECG, and ventricular pacing or pacemaker rhythm. The study was approved by the Ethics Committee of People's Hospital of Zhengzhou University and the Ethics Committee of Fuwai Central China Cardiovascular Hospital.

In total, 112 survivors of MVAEs (defined as VT/VF, syncope, or aborted sudden death) after out-of-hospital cardiopulmonary resuscitation and in-hospital cardioversion and defibrillation or patients who spontaneously converted to sinus rhythm after MVAEs and 173 patients with STEMI but without MVAEs were admitted to the catheterization room. Five patients died of hemodynamic collapse after PCI. All patients received heparin (5000 U) before angiography. Coronary angiography was performed using the standard Judkins technique. Before PCI, an additional dose of heparin (100 U/kg) was administered intravenously. Coronary balloon angioplasty or coronary stent implantation was performed in all patients. The intervention was considered successful when the residual stenosis was reduced to < 25% with a TIMI grade of 3. Patients who underwent coronary stent implantation were initiated on a loading dose of dual antiplatelet drugs namely, ticagrelor (180 mg) and aspirin (300 mg).

**Fig. 1:**
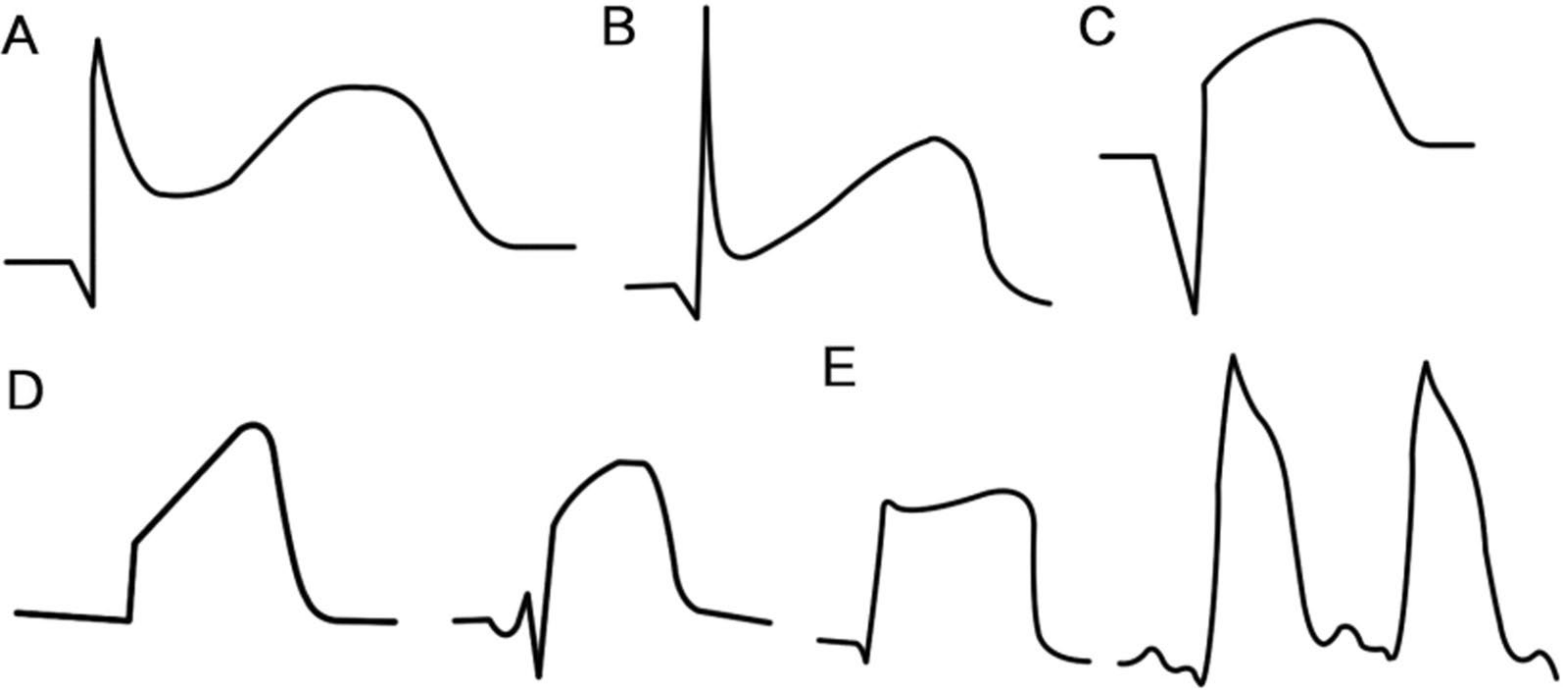
In the acute phase of ST-segment elevation myocardial infarction, ST-segment elevation has five morphologies: **A** arcuate downward type (crescent type), **B** oblique straight type, **C** arcuate upward type, **D** tombstone type, and **E** lambda-wave, giant R waves, or triangular QRS-ST-T waveform

From the onset of chest pain, all ECG records before and after admission were used in the analysis. The ECGs were recorded with a standard digital recorder using 12 simultaneous leads at a paper speed of 25 mm/s. STE and MVAEs were detected and recorded by ECG when the symptom onset and MVAEs were correlated with STE. In the acute phase of STEMI, STE has five morphologies: (A) arcuate downward type (crescent type), (B) oblique straight type, (C) arcuate upward type, (D) tombstone type, and (E) lambda-wave, giant R waves, or triangular QRS-ST-T waveform (Fig. 1). A single ECG lead showing maximum STE was reviewed. In anterior and non-anterior leads showing STE, the greatest STE from a single lead was measured from the base to point 40 ms after the J point. If STE and T waves on ECGs were fused into a monophasic curve or giant R-wave and the pattern was identified as a J point, the case was excluded. In cases where Tp-e interval was difficult to identify due to the huge R-wave pattern, Tp-e interval was not measured to highlight the significance of STE in MVAEs and effectively draw comparisons between the groups. The STE was manually measured according to the procedure described in a previous study [7]. The reported value for STE in all leads was the maximum obtained by two independent experts. In case where there is a difference of 0.5 mV in each measurement, a third expert was recruited. Each parameter was measured by averaging three consecutive beats. The interobserver Pearson correlation coefficient for STE was 0.963.

Continuous variables were expressed as mean ± standard deviation and, if appropriate, were compared using the dependent-samples Student t-test. Categorical variables were expressed as number and percentage and, if appropriate, were compared using the chi-square test. Binary logistic regression analysis was performed to assess the association of each predictor with the outcome, and further multivariable logistic regression analysis was conducted to estimate the association of STE with MVAEs after controlling for other factors. Analyses of receiver operating characteristic (ROC) curves were performed to examine the prognostic value of ECG parameters and determine cut-off values. The optimal cut-off value was defined as the value yielding the maximal Youden index (Youden index = Max ([sensitivity] + [specificity] − 1)) or the best combined sensitivity and specificity. Statistical analysis was performed using SPSS 20.0.0 (IBM Corp., Armonk, NY). Areas under the curve (AUC) were compared using MedCalc 15.2.2 (MedCalc Software, Ostend, Belgium) with the *Z* test. A *p* value < 0.05 was deemed statistically significant.

## Results

Overall, 16 patients were excluded from this study for the following reasons: obscure ECGs due to left or right bundle branch block (7 patients) and ventricular pacing or pacemaker rhythm (9 patients). In total, 285 patients were included in this study with clinical characteristics and risk factors for coronary artery disease presented in Table 1. We classified 173 patients into the non-MVAE group (127 men and 46 women; mean age, 57 years) and 112 into the MVAE group (93 men and 19 women; mean age, 57 years). There were no differences in the following characteristics: age, sex, hypertension, diabetes mellitus, smoking status, left ventricular ejection fraction, left ventricular end diastolic diameter, blood potassium concentration on admission, or infarct-related artery between the two groups (all, *p* > 0.05). However, the time from symptom onset to revascularization was more delayed in the MVAE group than in the non-MVAE group (*p* < 0.05), and the elevated ST-segment on ECG was significantly higher in the MVAE group than in the non-MVAE group (*p* < 0.05).

**Table 1:** Baseline clinical characteristics of study patients with and without MVAEs

	Non-MVAE group (n = 173)	MVAE group (n = 112)	*p* value
Age, years	57.23 ± 15.9	57.87 ± 13.2	0.7148
Male sex, n (%)	127(73.4%)	93(83%)	0.0806
Symptom-to-balloon time, hours	2.79 ± 1.79	2.35 ± 1.42	0.0285
Hypertension, n (%)	79(45.7%)	58(51.8%)	0.3741
Diabetes mellitus, n (%)	75(43.4%)	49(43.7%)	0.9552
Smoker, n (%)	70(40.5%)	47(42%)	0.8978
Left ventricular ejection fraction (%)	57.72 ± 11.6	58.29 ± 12.15	0.176
Left ventricular end diastolic diameter (mm)	46.79 ± 18.5	46.31 ± 19.6	0.3735
Blood potassium concentration at admission (mmol/L)	4.04 ± 0.34	4.01 ± 0.38	0.266
*Infarct-related artery*			
Left anterior descending artery, n (%)	51	41	
Right coronary artery, n (%)	96	53	0.0867
Left circumflex artery, n (%)	23	11	
Left main artery, n (%)	3	7	
STE max (mV)	3.38 ± 1.27	6.62 ± 2.84	*p* < 0.0001

STE max = maximal ST-segment elevation in the lead with the most prominent elevation, MVAE = malignant ventricular arrhythmia event

Clinical characteristics and ECG parameters were analyzed using a binary logistic regression analysis with the enter method, and the factors associated with a significantly increased risk for MVAEs during the acute early phase were shown in Table 2. Coronary arteries were then divided into six categories (left anterior descending artery [LAD], right coronary artery [RCA], left circumflex artery [LCX], left main artery [LM], double vascular lesions, and multiple vascular lesions), and LAD was considered a dummy variable for the classification of the lesion vessels. Binary logistic regression analysis was performed, and symptom-to-balloon time, age, STE, and LM lesion were included in the model. A stepwise regression analysis was then performed wherein symptom-to-balloon time, age, and STE were noted to be associated with MVAEs. Vascular lesions were then divided into four vessel classifications, while double vascular lesions and multiple vascular lesions were merged into the infarct-related artery. The symptom-to-balloon time, age, STE, LM lesion, and RCA lesion were included in the model. After stepwise regression analysis, only the symptom-to-balloon time, age, and STE were noted to be associated with MVAEs. Finally, the three coronary arteries (LAD, RCA, and LCX) were analyzed in the logistic regression analysis. In both the enter and stepwise methods, only STE was significantly associated with the occurrence of MVAEs (Table 2; all, *p* < 0.01).

**Table 2:** Multivariate regression model for predicting MVAEs

	Enter method	*p* value	Stepwise method	*p* value
	OR (95% CI) for MVAEs		OR (95% CI) for MVAEs	
Six vascular classifications Symptom-to-balloon time	0.7311 (0.574–0.9311)	0.0111	0.7819 (0.221–0.829)	0.035
Age	1.0779 (1.0024–1.0541)	0.0318	1.0292 (1.0054–1.0537)	0.0161
STE	3.4591 (2.5352–4.7197)	< 0.01	3.4591 (2.5352–4.7197)	< 0.01
LM	15.0038 (1.4889–151.1995)	0.0216		
*Four vascular classifications*				
Symptom-to-balloon time	0.7502 (0.5929–0.9492)	0.0166	0.7819 (0.6221–0.9829)	0.035
Age	1.0323 (1.0017–1.0580)	0.0116	1.0292 (1.0054–1.0537)	0.0161
STE	3.5577 (2.5885–4.8898)	< 0.01	3.1761 (2.3805–4.2375)	< 0.01
LM	11.5020 (1.3289–99.5538)	0.0265		
RCA	2.7871 (1.2180–6.3774)	0.0152		
*LAD classification*				
STE	3.1301 (1.8645–5.2547)	< 0.01	2.6252 (1.7362–3.9694)	< 0.01
*RCA classification*				
STE	4.0622 (2.4844–6.6421)	< 0.01	3.8692 (2.4433–6.1274)	< 0.01
*LCX classification*				
STE	10.9212 (1.9589–60.8863)	< 0.01	9.1880(2.0147–41.9017)	< 0.01

*MVAE* malignant ventricular arrhythmia event, *OR* odds ratio, *CI* confidence interval, *STE* ST-segment elevation, *LM* left main artery, *RCA* right coronary artery, *LAD* left anterior descending artery, *LCX* left circumflex artery

ROC curve analysis was performed to assess the sensitivity, specificity, and best cut-off values for STE to predict MVAEs in different infarct-related arteries (Table 3). The AUC of STE for predicting MVAEs was 0.905, and the cut-off value was 4.5 mV, with the best combination of sensitivity and specificity (81.25% and 90.17%, respectively) when all infarct-related arteries were included in the analysis. The AUC of the LAD was 0.925 with a cut-off value of 4.5 mV, that of the RCA was 0.915 with a cut-off value of 4.5 mV, and that of the LCX was 0.929 with a cut-off value of 4.0 mV.

**Table 3:** AUCs and cut-off values of STE for predicting MVAEs

	AUC	Cut-off value	Sensitivity (%)	Specificity (%)	*p* Value
All coronary arteries	0.905 (0.865–0.936)	> 4.5	81.25	90.17	< 0.001
LAD	0.925 (0.851–0.970)	> 4.5	97.56	78.43	< 0.001
RCA	0.916 (0.859–0.955)	> 4.5	71.7	93.75	< 0.001
LCX	0.929 (0.786–0.988)	> 4	81.82	95.65	< 0.001

*MVAE* malignant ventricular arrhythmia event, *RCA* right coronary artery, *LAD* left anterior descending artery, *LCX* left circumflex artery, *AUC* area under the curve

## Discussion

The main findings of this study were as follows: (1) the symptom-to-balloon time, age, and STE were associated with MVAEs before and during reperfusion in STEMI, and (2) in patients with STEMI presenting in the first 6 h from symptom onset, STE was the strongest independent predictor for MVAEs among the factors studied.

About 64% of VT/VF episodes occur before and during PCI, and 90% of patients exhibit symptoms of STEMI within 48 h [11]. Although some investigations have suggested that VT/VF occurring during PCI does not correlate with clinical outcomes, more trials have demonstrated an increased incidence of in-hospital and out-of-hospital adverse prognosis before and during PCI as well as 3-year mortality prior to revascularization in patients with MVAEs [12, 13]. Furthermore, malignant outcomes are worse if VT/VF occurs late instead of early [5, 11]. Fortunately, the recent investigation shows the use of sodium-glucose co-transporter 2 inhibitors (SGLT2-i) was associated with a lower risk of new-onset arrhythmic events, especially VT/VF, during hospitalization and long-term follow-up for AMI patients complicated T2DM [14, 15]. Therefore, the difference in the effect of VT/VF on the prognosis of STEMI shows the varied electrophysiological mechanisms of VT/VF.

An MVAE is thought to be caused by electrophysiological abnormalities of ventricular repolarization. In cellular models, the extent of STE represents a potential gradient for phase 2 re-entry, which is the primary mechanism underlying arrhythmogenesis in patients with Brugada syndrome and STEMI [16]. Therefore, STE may be the main predictor of malignant ventricular arrhythmia in patients with STEMI in the early phase (such as that occurring before and during PCI). Some investigations have shown that presentation within 6 h of symptom onset is an independent predictor of sustained VT/VF, wherein ST segment might have the highest elevation [11, 17]. After 6 h, regardless of whether reperfusion treatment has been employed or not, STR reached a certain extent and is accompanied by pathological Q wave and reversed T wave. This indicates relatively low incidence of VT/VF events. Therefore, the appropriate cut-off time for predicting MVAEs in the early phase in patients with STE should be within 6 h of symptom onset. Moreover, a considerable proportion of VT/VF before PCI might be induced by spontaneous vascular recanalization, which is consistent with the mechanism of ventricular arrhythmia during PCI. Therefore, in this study, we selected STEMI patients within 6 h of symptom onset to study the predictors of malignant arrhythmia in the early stage of STEMI (before and during PCI).

Our study showed that after using the entry method, an earlier symptom-to-balloon time, LM and RCA lesions, older age, and greater STE were related to a higher occurrence of MVAEs as evidenced in the multivariate regression analysis. However, after stepwise analysis, the occluded vascular site did not enter the regression model. Multivariate regression analysis of a single vessel showed that STE was a risk factor for predicting malignant ventricular arrhythmia. Previous studies have shown the following risk factors for MVAEs namely, no previous history of MI or angina pectoris, LM lesion, inferior MI, and Tp-e interval of infarct-related leads [9, 10]. In this study, patients with a history of previous MI or angina pectoris were excluded from this study to eliminate the influence of ischemic preconditioning and the establishment of collateral circulation caused by previous ischemic history. In some studies, older patients were less likely to develop malignant arrhythmias [18], since the ischemic myocardium of older patients had collateral circulation or an ischemic preconditioning function caused by a history of angina pectoris or MI resulting in non-STEMI or lower STE. Consequently, the incidence of malignant ventricular arrhythmia was also low. Age was positively correlated with the occurrence of malignant ventricular arrhythmia, which is consistent with the results of other studies [19]. The entry method also revealed that LM disease or inferior MI can also be used as a predictor of MVAEs while double-vessel and multivessel lesions could not effectively predict MVAEs. The latter could be due to ischemic preconditioning or the establishment of collateral circulation induced by previous multiple lesions. We know that VT/VF caused by left main disease can be very severe. The higher incidence of VF in inferior STEMI, especially with right ventricular involvement, may be due to the more prominent transient outward current(Ito) in the epicardium of the right ventricle than that of the left ventricle [20]. However, the location of the vascular lesions could not be included in the model once symptom onset time and STE are present. This indicates that the time to symptom onset and STE were stronger predictors of MVAEs. In some patients in this study, the Tp-e interval could not be identified and measured during the early phase of ECG; hence, it was not included. A previous meta-analysis showed that when factors without STE were involved, the time to onset of symptoms (i.e., earlier admission time) could predict MVAEs more than hypertension, diabetes, sex, and other risk factors. However, STE was more strongly associated with VT/VF than with earlier admissions [21].

In previous studies, we discussed the mechanism of STE and its possible relationship with the Tp-e interval [7, 22]. In the acute phase of STEMI, STE has five morphologies. In ventricular wedge preparation, global ischemia could cause an all-or-none repolarization at the end of phase 1 of the action potential in the epicardium, but not in the endocardium, leading to STE and early after-depolarization activity secondary to phase 2 re-entry; this results in a tombstone configuration in the ECG. Although the mechanism of STE and Tp-e interval formation is still controversial, we believe that STE was caused by the difference between the duration and amplitude of action potential in the infarcted and non-infarcted areas. Tombstone type and giant R waves on ECG were special manifestations of STE, which indicated that the duration and amplitude of action potential in the infarct area were extremely reduced or even disappeared, and the morphology of ECG only showed the action potential pattern of the non-infarct area. Thus, tombstone type and giant R waves represented the maximum degree of STE, while the amplitude of ST-segment elevation and the length of Tp-e interval represented potential gradient dispersion of phase 2 re-entry, which was the main factor inducing early after-depolarization. Early after-depolarizations were considered as the underlying mechanisms of VT/VF [22]. Patients with tombstone type and giant R waves on ECG or significant STE were more prone to MVAEs than those without such characteristic ECG changes. Therefore, STE reflects repolarization potential dispersion as well as predicts MVAEs and in-hospital mortality [4, 23].

In terms of pathological mechanisms, ST elevation is a manifestation of segmental myocardial damage. The severity of ischemia during acute MI, reflected by the magnitude of STE, was influenced by the magnitude of collateral circulation supplying the infarcted area. The establishment of collateral circulation indicated a history of ischemia and thus had ischemic preconditioning. This can lead to a lower STE, a smaller repolarization potential gradient, and smaller MI area, which ultimately prevents the occurrence of malignant arrhythmia and reperfusion arrhythmia in the acute stage. This also predicts the preservation of cardiac function. Patients with tombstone type and giant R waves showed maximal regional ischemia without collateral circulation and were prone to developing MVAEs [7, 19]. The presence of STR after both pharmacological and mechanical reperfusion therapy is highly predictive of infarct-related artery patency and the degree of effective microvasculature perfusion, which was inversely related to infarct size. Thus, STR was shown to be a significant independent predictor of major adverse cardiovascular events and target vessel revascularization in short- and long-term follow-ups [8, 24]. STE represented the size of the acute infarcted myocardial area and the resulting potential gradient in the infarcted and non-infarcted regions leading to malignant arrhythmia. Meanwhile, STR reflected the number of recovered infarcted myocardium, which indicates the preservation of myocardial function and long-term prognosis.

### Study limitations

The study has several limitations. First, to highlight the role of STE in predicting MVAEs in patients with early STEMI, we conditionally screened patients in the MVAE group and the control group, specifically those without a history of MI or angina pectoris, within 6 h of admission. Consequently, the study results cannot be extrapolated to all patients, although it does not affect the reliability of the clinical significance of the ST segment; that is, in the acute stage, STE predicts the amplitude of the potential gradient that plays an important role in the occurrence of ventricular arrhythmia. Moreover, because of the limited conditions, only few patients were enrolled in the study as well as those patients with LM disease. There was no significant difference in age and criminal blood vessels between the MVAE and non-MVAE groups in the chi-square analysis. Multivariate analysis showed that age and lesions arteries could be entered into the regression model. If the number of sample patients is increased, the effectiveness of chi-square analysis and multivariate regression analysis can be significantly improved.

## Conclusions

In patients with STEMI presenting within 6 h of onset, age, symptom-to-balloon time, and STE were the main predictors for MVAEs, and among which, STE was the strongest predictor. In the acute stage, STE was an index reflecting repolarization dispersion of the action potential of cardiomyocytes in infarcted and non-infarcted areas.

## Data Availability

Part of the datasets used and/or analyzed during the study are available from the corresponding author upon reasonable request.

## Abbreviations

STE: ST-segment elevation
AMI: Acute myocardial infarction
STEMI: ST-segment elevation myocardial infarction
MVAE: Malignant ventricular arrhythmia event
AUC: Area under the curve
SCD: Sudden cardiac death
VT: Ventricular tachycardia
VF: Ventricular fibrillation
ECG: Electrocardiography
PCI: Percutaneous coronary intervention
STR: ST-segment resolution
TIMI: Thrombolysis in myocardial infarction
ROC: Receiver operating characteristic
LAD: Left anterior descending artery
RCA: Right coronary artery
LCX: Left circumflex artery
LM: Left main artery
SGLT2-i: Sodium-glucose co-transporter 2 inhibitors
T2DM: Type 2 diabetes mellitus
